# Supplementary material for: Polio vaccination and marginal effects among children aged 12–23 months in Lesotho using 2024 LDHS data: A multilevel analysis approach
Source: PLoS One. 2025 May 6;20(5):e0322731. doi: 10.1371/journal.pone.0322731 (PMC12054887; doi:10.1371/journal.pone.0322731)
Supplement: Attachment — Submitted filename: Response to Reviewers.docx [file pone.0322731.s004.docx]

Date: 27 Jan 2025

**Response Letter to the Editor and reviewer (s) Comments**

**JOURNAL: PLOS ONE**

**Respected Editor and reviewer(s)!**

We would like to thank you very much for your professional review and scientifically sounded comments which are very relevant to improve our manuscript entitled “Polio vaccination and marginal effects among children aged 12-23 months in Lesotho using 2024 LDHS data: A multilevel analysis approach"

We are glad about the given comments and accepted your suggestions and comments and revised the manuscript accordingly as described below.

I. **Response to the comments about Journal requirements**
1. Please ensure that your manuscript meets PLOS ONE's style requirements, including those for file naming.

**Response: Dear(s),** thank you for the comments and suggestions about the PLOS ONE Journals requirements!

The manuscript has been edited based on all the requirements like font size, bolding and file naming….

2. In the online submission form, you indicated that [The data will available by the corresponding author/DHS program website upon request.].

- All PLOS journals now require all data underlying the findings described in their manuscript to be freely available to other researchers, either 1. In a public repository, 2. Within the manuscript itself, or 3. Uploaded as supplementary information.

**Response:** Thank you very much about your comments about data availability statement! We revised and rephrased the data availability statement on both the manuscript and the portal system as:

“The *data used in this study is available freely online from DHS programs website i.e (*[*https://www.dhsprogram.com/data*](https://www.dhsprogram.com/data)*)”.*

II. **Response to the comments from the attached manuscript**

1. About **Marginal effects** on the title

This reads like a conclusive term – It could be changed based on this statement in the last paragraph of the Introduction

**Response:** Thanks for your constructive comments.

- The objective of this study is evaluating the polio vaccination variability between clusters and indicating **marginal effects** of variables on each category of polio vaccination.
- We used the **marginal effects** on the title section for a reason of indicating this marginal effects analysis is one part of the study objective, so we think that this statement was not considered as conclusion.

2. Comments related to the reference citations and abbreviations on the manuscript?

**Response:** Thank you for your useful constructive comments.

We really appreciate for the comments and the manuscript has been revised (inserted citation and we wrote the full name of the abbreviations) according to the comments.

3. About the following statement from last paragraph of the introduction

“Additionally, the analysis showed the marginal impacts of each explanatory variable on polio vaccination status categories”.

- Is this a conclusion/result of this study - if so, it should be moved from the introduction.

**Response: Thank you very much for your comments here.**

- We used this statement NOT as conclusion rather was one part of the objective of the study because this study indicted the marginal effects of independent variables. We re-write this statement as one of the parts of the objective of the study (see last paragraph of the introduction section).

4. Comments on the first paragraph on Methods section.

This information may not be very relevant in the method - maybe as part of the intro/discussion to give context

**Response: Thank you very much for your comments suggestions.**

Based on your suggestions we revised these statements and the revised statements here used to explain about:

- The type of data used for the study
- How the data was collected
- Who conducted the survey
- The sampling methods

So these are why and we believe these are related to the data which are important information in the methods section.

5. Comments from the statements: --------29 sampling strata were created based on????. In the first sampling stage, 400 EAs in each sampling stratum were selected independently with probability based on EA size. In the second round of selection, 25 households per cluster (EA)…

- Basis for 40??
- Basis for 25??

**Response: Dear (s), thank you very much for your valuable comments.**

As per the given comments, we revised this section and these are on the basis of “the 2016 Lesotho population and housing census”.

6. Comments about sample size

How was 529 derived

What was the target sample size and how was it determined. This is important to evaluate the power of the conclusions

**Response: Dear (s), we have great appreciate to you once again.**

- The target population for the study was all children in Lesotho within the age group of 12-23 months.
- The data used in this study was extracted from the Lesotho DHS data with polio vaccination status and there are **529 un-weighted numbers of children** in the specified age groups.

7. Comments about the paragraph in **multilevel ordinal regression model and marginal effects** section…

This reads like definitions of what multilevel ordinary regression model is. Describe what was done using this statistical analysis and not what it is ..

**Response: Dear (s), thank you again for this sounded comment.**

Based on the given constructive comment, we revised and re-write the manuscript by describing what was done using the statistical methodologies and statistical tests.

8. **Comments in result section**

--- about the sample size of 490??

**Response: Thank you very much dear(s).**

In this study there are 529 un-weighted number of children obtained from LDHS data. Since the data is DHS, we were weighting the data and the weighted sample (number of children) was 490, which was stated in the result sections.

9. About the percentage of this statement ……..aged 12 to 23 months, with 27 (5.5%) of them not vaccinated, 146 (29.7%) either not vaccinated or incompletely vaccinated, and 344 (70.4%) fully vaccinated

Report on the 3 categories in the table

**Response: Thank you very much dear(s).**

Yes, there are 3 categories of the response (polio vaccination levels) and this response variable has an ordinal scale we used the cumulative prevalence of not vaccinated and incomplete vaccinated (see table 1).

10. Comments in the descriptive analysis.

**Response: Thank you very much dear(s).**

We accepted the comments and revised the manuscript based on the given comments (we added the percentages and missed statistics values including the table).

11. Comments about multilevel ordinal logistic regression mode

This can be used in response to my earlier comment- this is a description of method and not results

**Response: Thank you very much dear(s).**

Based on the given sounded comment, we revised and re-write the manuscript as a result description. The statements here described the results of some statistical tests and these are parts of the results.

12. Comments about discussion

**Response: Thank you very much dear(s).**

We revised some of the discussion sections of the manuscript according to the given comments.

13. Comments about the conclusion.

**Response: Thank you very much dear(s).**

**We revised the manuscript based on the given constructive comments in the conclusion section.**
