# Supplementary material for: Polio vaccination and marginal effects among children aged 12–23 months in Lesotho using 2024 LDHS data: A multilevel analysis approach
Source: PLoS One. 2025 May 6;20(5):e0322731. doi: 10.1371/journal.pone.0322731 (PMC12054887; doi:10.1371/journal.pone.0322731)
Supplement: Attachment — Submitted filename: Response_to_Reviewers_auresp_2.docx [file pone.0322731.s005.docx]

Date: 11 Feb 2025

**Response Letter to the Editor and reviewer (s) Comments**

**JOURNAL: PLOS ONE**

**Respected Editor and reviewer(s)!**

We would like to forward a great gratitude to you for your professional review and scientifically sounded comments used as a corner stone for improving our manuscript entitled “Polio vaccination and marginal effects among children aged 12-23 months in Lesotho using 2024 LDHS data: A multilevel analysis approach"

We have always a great appreciation for the given comments and we accepted your suggestions and comments then revised the manuscript accordingly as described below.

I. **Response to the comments about Journal requirements**
1. Please review your reference list to ensure that it is complete and correct. If you have cited papers that have been retracted, please include the rationale for doing so in the manuscript text, or remove these references and replace them with relevant current references. Any changes to the reference list should be mentioned in the rebuttal letter that accompanies your revised manuscript. If you need to cite a retracted article, indicate the article’s retracted status in the References list and also include a citation and full reference for the retraction notice.

**Response: Dear(s),** thank you for the comment and suggestion about the PLOS ONE Journals requirements!

All the references are checked for their correctness/completeness and we didn’t cite any retracted papers in the manuscript.

II. **Response to the comments from the attached manuscript**

1. About **DHS data description…**in methods section

This is still a description of another study, and not this study.

**Response:** Thanks for your constructive comments once again.

- Based on your given comment, we revised this section in the manuscript. See page 4-5 in the manuscript

2. About sample size of 529…….

How was the 529 derived - till not described/explained

Why not 600….or not 800..or not 100………….how did you arrive at 529

**Response:** Thank you very much dear editor!

We really appreciate for the comments and the manuscript was revised (we added the flow chart that showed how the sample of 529 children was derived from Kids file in LDHS data).
